# Preliminary regression-based feature selection for optimizing spatial frequency domain imaging in burn-severity assessment

**DOI:** 10.1117/1.JBO.31.10.106007

**Published:** 2026-10-30

**Authors:** Thinh Phan, Christopher A. Campbell, Gordon T. Kennedy, Nicole Wakida, Nataliya Makeeva, Gabriela Tabone, Robert J. Christy, Theresa L. Chin, Victor C. Joe, Bernard Choi, Anthony J. Durkin

**Affiliations:** a University of California – Irvine, Beckman Laser Institute and Medical Clinic, Irvine, California, United States; b University of California – Irvine, Department of Biomedical Engineering, Irvine, California, United States; c University of Texas – San Antonio, Military Health Institute, San Antonio, Texas, United States; d University of California – Irvine, Department of Surgery, Irvine, California, United States

**Keywords:** spatial frequency domain imaging, burn wound severity assessment, linear regression, feature selection, collagen, machine learning

## Abstract

**Significance:**

We identified key spatial frequency domain imaging (SFDI) features for assessing burn wound severity. We built upon our previous two-step regression framework, using SFDI-calibrated reflectance ($R_{d}$) maps to predict collagen denaturation depth (CDD) and healing outcome of burn wounds. We used feature selection methods to identify key parameters from a large data set (40 features, including eight wavelengths, each at five spatial frequencies).

**Aim:**

We aimed to identify a reduced set of key SFDI features (i.e., pairs of spatial frequencies and wavelengths) for assessing burn wound severity.

**Approach:**

We utilized a graded-severity porcine burn model (n=4) with eight burn contact durations (5 to 40 s). Multiple linear regression (MLR) was employed to predict CDD from $R_{d}$ obtained within 24 h post-burn. Subsequently, logistic regression (LogR) was used to predict day-28 healing outcomes based on the predicted CDD. In this study, we fielded three feature selection algorithms (i.e., standardized ordinary least squares, least absolute shrinkage and selection operator, and minimum redundancy maximum relevance) to identify key SFDI features in the MLR model. The resulting prediction then dictates the LogR outputs. We assessed the models for accuracy and fit performance against those of the 40-feature original model.

**Results:**

A subset of 13 LASSO features matched the predictive performance of the 40-feature baseline (RMSE 98 versus 105  μm; within fold-to-fold noise), and a minimum set of eight LASSO features (minimum features with acceptable errors) closely approximated it. Across all three methods, the consistently top-ranked features were planar ($0\;{\mathrm{mm}}^{-1}$) reflectance at 526, 591, and 621 nm.

**Conclusions:**

Using feature selection algorithms, we identified preliminary candidate features that may be suitable for developing a future SFDI system to assess burn wound severity with reduced complexity. This has the potential to enable a more compact design compared with the current research-grade SFDI system.

## 1 Introduction

Burn injuries can happen to anyone, anytime, and anywhere.^1^^–^^4^ Severe burns often produce scars that are cosmetically and functionally compromising. Early and accurate triage is crucial for maximizing healing. The current standard of practice involves clinical diagnosis by burn surgeons to determine the need for debridement and grafting. This approach is inherently prone to subjective interpretations.^5^^–^^8^ In fact, experienced surgeons are only 60% to 75% accurate in determining burn severity, specifically in distinguishing superficial partial thickness burns, which can heal with appropriate wound care, from deep partial thickness burns, which require debridement and often grafting.^9^^,^^10^

Advances in imaging techniques have enabled a more objective assessment of burn severity.^8^^,^^10^^–^^15^ One such technique is spatial frequency domain imaging (SFDI). SFDI is a wide-field imaging modality that utilizes structured illumination, often in combination with a model of light propagation, to extract the tissue’s absorption and scattering properties.^16^^–^^20^ These properties are associated with functional and structural information, respectively.^16^^,^^17^ Our previous studies suggest that SFDI can aid in differentiating burn severities, including distinguishing superficial partial thickness burns from deep partial thickness burns.^21^^–^^24^

This paper builds upon a two-step regression framework we recently developed.^25^ The framework leverages SFDI-calibrated reflectance ($R_{d}$) maps to evaluate the depth of completely denatured collagen in burn wounds. Acknowledging denatured collagen as a hallmark of thermally damaged skin,^26^^–^^28^ we showed that collagen denaturation depth (CDD) can predict burn severity and subsequent healing outcomes through two regression steps.^25^ Step 1 uses multiple linear regression (MLR) with day-1 $R_{d}$ to predict day-1 CDD, and step 2 uses logistic regression (LogR) with day-1 CDD as input and day-28 healing outcome as output. However, the framework employed eight wavelengths (i.e., 471, 526, 591, 621, 659, 691, 731, and 851 nm) and five spatial frequencies (i.e., 0, 0.05, 0.1, 0.15, and $0.2\;{\mathrm{mm}}^{-1}$) for a total of 40 SFDI reflectance images. This large wavelength-frequency space led to extended acquisition times and thus increased susceptibility to motion artifacts such as breathing.

To advance the clinical translation of SFDI for burn wound assessment, we aim to simplify the input requirement for this framework. Specifically, our goal is to identify a reduced subset of diagnostically relevant wavelengths and spatial frequencies, herein referred to as SFDI “features.” In the machine learning literature, this process is called “feature selection” (FS) and is widely used in multispectral or hyperspectral imaging modalities.^29^^–^^32^ Our previous study used a rudimentary feature-selection approach in which we included only planar $R_{d}$ maps (i.e., spatial frequency of $0\;{\mathrm{mm}}^{-1}$) to train a cubic support vector machine for burn-severity classification. This planar-only model showed reduced performance compared with models that incorporated high-spatial-frequency information.^24^ Although this result underscores the importance of combining low and high spatial frequencies, it remained unclear which specific wavelength–frequency combinations were required.

An exhaustive search of all possible SFDI feature combinations is computationally impractical. In the current study, we therefore applied three established FS methods to identify key SFDI features in predicting CDD: (1) standardized ordinary least squares (SOLS) linear regression, (2) least absolute shrinkage selection operator (LASSO), and (3) minimum redundancy maximum relevance (mRMR). Each method yielded streamlined models with fewer features, which we compared against the baseline 40-feature model. The final reduced-feature model identifies key SFDI features that may simplify future system design and shorten acquisition time, supporting translation to environments requiring a compact, rapid burn triage device.

## 2 Approach Overview

Our group focuses on applying SFDI, together with a range of analytical methods, to assess burn severity. Historically, we captured SFDI data using the Reflect RS™ (Modulim, Irvine, California, United States) at eight wavelengths and five spatial frequencies (i.e., values relevant to the extraction of optical properties).^18^^,^^33^^–^^36^ The SFDI processing workflow involves imaging a phantom with known optical properties to determine the instrument response function and generate calibrated reflectance ($R_{d}$).^16^^,^^17^^,^^19^

We previously showed that a cubic support vector machine can classify burn depth categories directly from $R_{d}$ maps.^24^ Although effective, this machine-learning approach functions as a “black box,” limiting insight into how individual features contribute to predictions and making feature simplification or selection difficult. To address this, we returned to a more interpretable framework and developed a multiple linear regression (MLR) model to predict collagen denaturation depth (CDD) from SFDI $R_{d}$ data. MLR provides transparent regression coefficients that reveal how each SFDI feature influences the model’s outcome.^25^

In MLR models with many predictors, redundancy is common, either because a predictor has no linear relationship with the response or because it is linearly dependent on other predictors. Initial analysis of regression coefficients helped identify influential features, but a more rigorous FS strategy is needed.^25^ Diffuse reflectance data are unevenly distributed across spatial frequencies, with higher frequencies producing substantially lower reflectance values that can artificially inflate regression coefficients. Our current work focuses on more robust FS methods that address these imbalances, including standardization,^37^ regularization,^38^ information-theoretic metrics,^39^ and bootstrapping.^37^

As in the first study, we implemented the same two-step framework integrating MLR and LogR to assess burn depth and healing outcomes (Fig. 1).^25^ We then employed three FS methods to determine the most effective and efficient MLR models: (1) standardized ordinary least squares (SOLS), (2) least absolute shrinkage and selection operator (LASSO), and (3) minimum redundancy maximum relevance (mRMR).

**Fig. 1:**
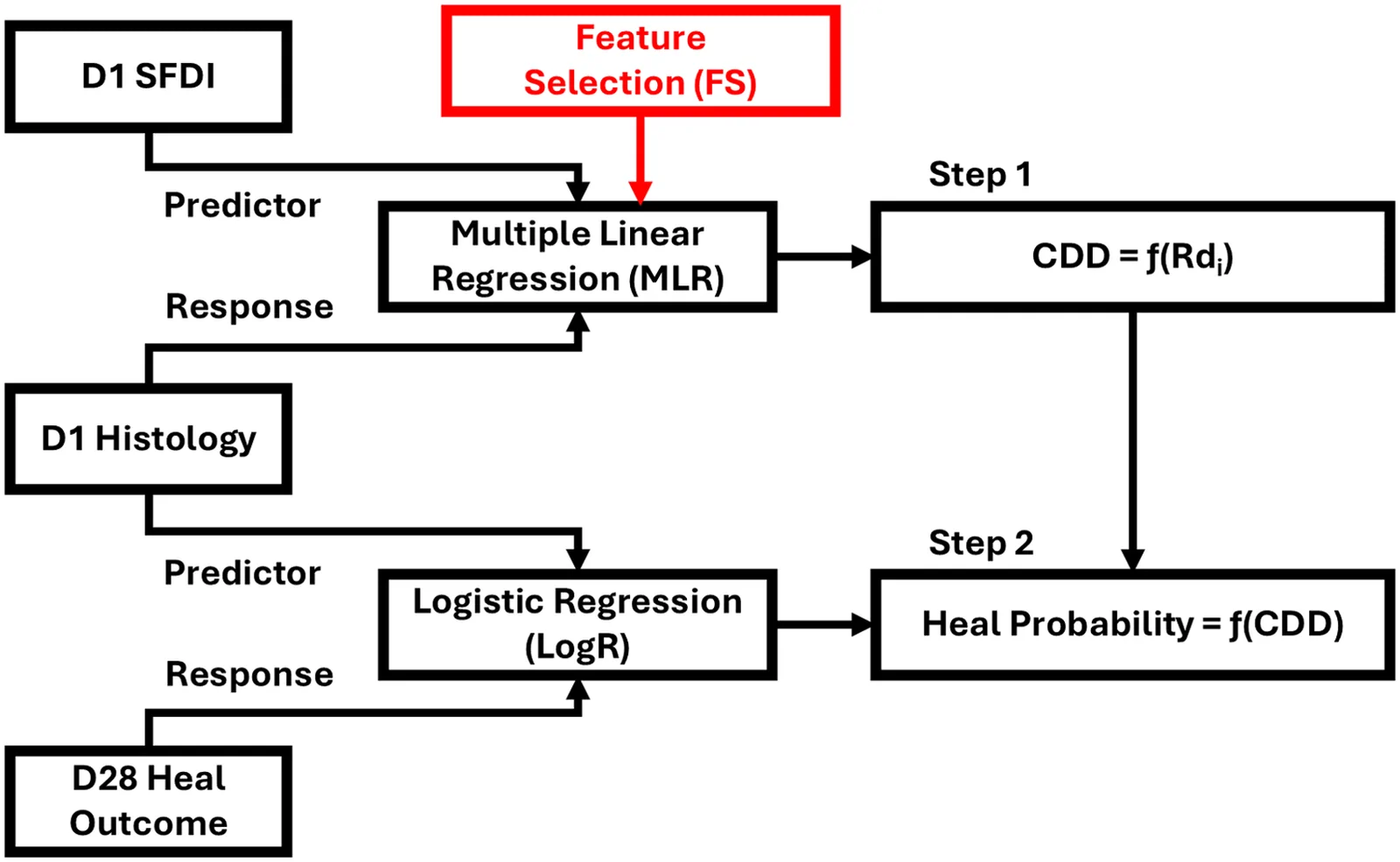
Overall workflow of the two-step regression analysis performed is similar to that of our prior work,^25^ with feature selection (FS) methods added. Step 1: Multiple linear regression (MLR) with FS was used to develop a model predicting histology-derived collagen denaturation depth (CDD) from calibrated reflectance ($R_{d}$). Step 2: Logistic regression (LogR) was applied to create a probabilistic and classification model of healing outcomes based on CDD.

In biomedical spectroscopic studies, such as diffuse reflectance spectroscopy, FS is often used to identify key wavelengths for specific research questions: for example, differentiating orthopedic tissues,^32^ measuring glucose and lactate concentrations,^30^ and diagnosing colorectal cancer.^29^ In this study, applying FS methods within the MLR framework allowed us to quantify the contribution of each SFDI feature ($R_{d}$ at a given wavelength–spatial-frequency pair) to CDD estimation.

## 3 Materials and Methods

### 3.1 Experimental Design

We described the experimental design in the previous publication.^25^ In summary, we employed an established porcine burn model in hybrid Yorkshire-Landrace pigs (n=4; two males and two females).^24^ Burns of varying severity were induced using a brass rod heated to 100°C and placed in contact with skin for 5, 10, 15, 20, 25, 30, 35, and 40 s along the dorsum on either side of the spine. SFDI and conventional color photography were performed on days 0, 1, 3, 7, 14, 21, and 28. Biopsies were collected on days 1, 7, 14, 21, and 28, and histology was assessed for collagen denaturation depth (CDD) using Picrosirius Red staining and polarization microscopy.^25^^,^^40^ We applied the new analysis method described in this study to the dataset previously described in Ref. 25. We obtained $R_{d}$ maps using a standard three-phase demodulation scheme^17^^,^^19^ together with a silicone-based calibration phantom supplied with the commercial SFDI system used in this study.

All procedures were carried out under UCI IACUC protocol AUP-24-056. Research was conducted in compliance with the Animal Welfare Act, the implementing Animal Welfare regulations, and the principles of the Guide for the Care and Use of Laboratory Animals. The facility where this animal research was conducted is fully accredited by the AAALAC International. Any opinions, findings, conclusions, or recommendations expressed in this material are those of the authors and do not necessarily reflect the views of the organizations above.

### 3.2 Data Sampling and Preparation

$R_{d}$ images were stored as an image cube (image width × image height × feature index), where the feature index corresponds to calibrated reflectance values at various spatial frequencies and wavelengths. Table 3 in Appendix B displays the full feature list with the corresponding wavelengths and spatial frequencies. A cropped image (221×221  pixels, or 6×6  cm) of the burn and the immediately surrounding tissue was used for further analysis. $R_{d}$ data were sampled from the biopsy site and from adjacent unburned skin using circular regions of interest (ROIs) with a 10-pixel (∼2.73  mm) radius (Fig. 2). The ROI size was selected to closely approximate the biopsy dimensions. ROI placement was determined through visual registration of the SFDI $R_{d}$ images with the corresponding color images.^25^

**Fig. 2:**
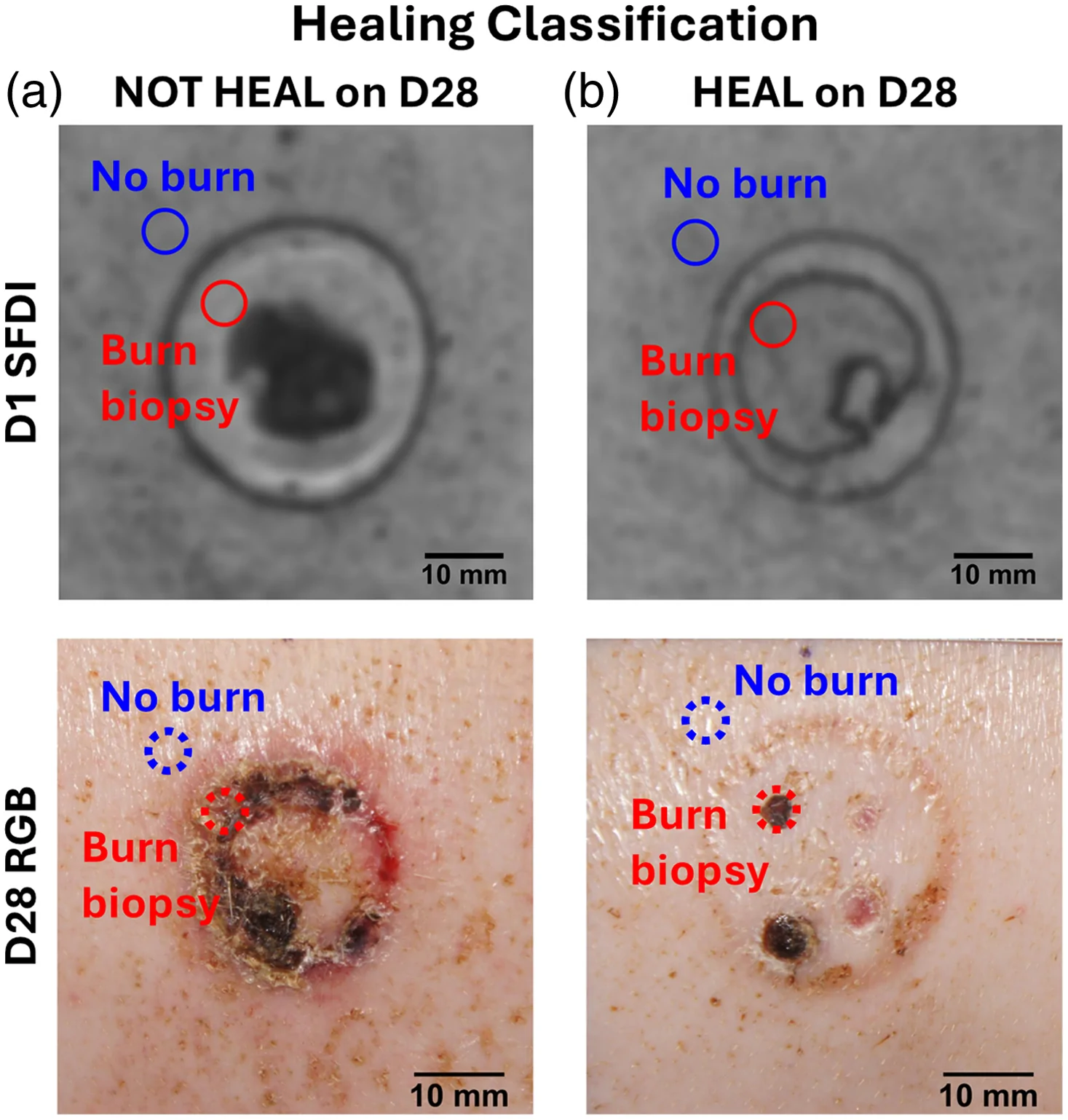
Representative $R_{d}$ images of chosen ROIs (red circles—burn, blue circles—no burn) for burns that did not heal (a) and that did heal (b) by day 28 (D28) as adapted from Ref. 25.

For histology, day-1 CDD was assessed using PSR-stained biopsy images from polarization microscopy.^25^^,^^35^ CDD was defined as the depth at which collagen was completely denatured and appeared dark (see Phan et al.’s^25^ figure 1). For each PSR microscopy image, 50 semi-automated CDD measurements were obtained, and the median value was used for subsequent analysis (see Phan et al.’s^25^ figure 4).

Day-28 healing outcomes were determined from conventional color photographs, consistent with prior work.^24^ Because biopsy samples were collected on day 1, the exact biopsy site was not available for visual evaluation on day 28. However, because biopsies were taken from visually homogeneous regions, we assumed that the surrounding tissue provided a representative measure of the healing status of the biopsied area.

### 3.3 Application of Feature Selection Methods in Multiple Linear Regression

In our previous work, we used an MLR model in which day-1 $R_{d}$ measurements at eight wavelengths and five spatial frequencies served as predictors of day-1 CDD.^25^ In the current study, we applied FS methods to identify the most informative SFDI features. Mathematical terminology, notation, and definitions are provided in Appendix A.

#### 3.3.1 Generation of a baseline general multiple linear regression model using all 40 SFDI features

We constructed the baseline general MLR model from all 40 SFDI features using ordinary least squares (OLS). As reported in the first study, model performance was evaluated using leave-one-subject-out cross-validation (LOSO CV) with metrics such as root mean square error (RMSE) and adjusted $R^{2}$.^37^

#### 3.3.2 Creation of rank-ordered lists of important features using three FS methods

To assess feature importance, we applied three FS methods: (1) standardized ordinary least squares (SOLS) linear regression, (2) least absolute shrinkage and selection operator (LASSO),^38^ and (3) minimum redundancy maximum relevance (mRMR).^39^ Each method produces a rank-ordered list of features based on their contribution to the overall MLR model. We performed 500 rounds of bootstrapping^37^ using half the dataset to calculate each feature’s average importance quotient (IQ), defined as the normalized ratio of a feature’s importance score to that of the most important feature identified. Because SOLS, LASSO, and mRMR derive importance from fundamentally different criteria, the IQ values reported here are within-method only and should not be compared directly across methods. The overall workflow is illustrated in Fig. 3. Details of each FS method are provided in Appendices A and B.

**Fig. 3:**
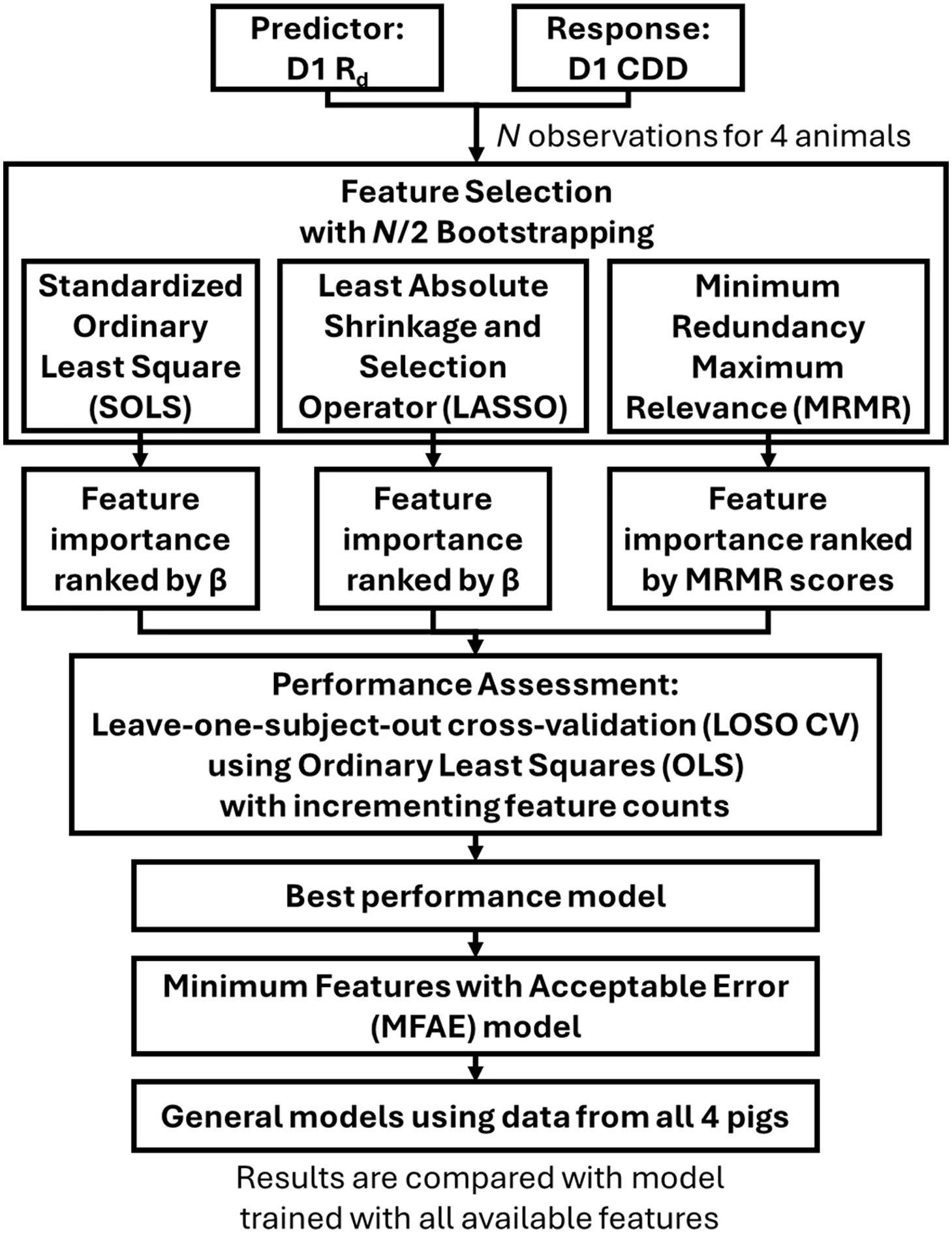
Workflow for multiple linear regression (MLR) with feature selection (FS): the predictor variable is SFDI-calibrated reflectance ($R_{d}$) from day 1, whereas the response variable is histology-derived collagen denaturation depth (CDD) from day 1. FS was conducted with bootstrapping to ensure robust importance rankings. Performance was evaluated using leave-one-subject-out cross-validation (LOSO CV) to generate output models.

#### 3.3.3 Generation of feature-reduced models using importance quotient lists

Our objective was to develop a feature-reduced MLR model with performance comparable to or better than the 40-feature model. We did this by incrementally increasing the number of features used in OLS based on the importance-ranked lists, evaluating each model using LOSO CV. We calculated the mean RMSE and adjusted $R^{2}$ across all LOSO CV folds for each feature count. The best-performing model and its corresponding FS method were identified as those with the lowest mean RMSE.

To evaluate the robustness of the key features, we defined a 5% error margin relative to the minimum mean RMSE, equivalent to ∼5  μm of CDD, a difference that is deemed histologically negligible for assessing burn severity by our burn experts. Using this margin, we identified the minimum feature set yielding acceptable error, termed the minimum features with acceptable errors (MFAE) model. All general models were then trained using data from all four animals and subsequently applied to every pixel within the FOV to generate full CDD maps. The performance of these feature-reduced models was compared with the baseline 40-feature model.

### 3.4 Probability and Classification Models of Healing Outcomes from Histological Collagen Denaturation Depth Using Logistic Regression

We employed the same logistic regression analysis described in the first study.^25^ In summary, we generated probabilistic and classification models using LogR with day-1 biopsy-derived CDD as the predictor and day-28 healing outcome as the response. We used a simple logit link function and evaluated the model using LOSO CV, with the area under the receiver operating characteristic curve (AUC ROC) calculated for both training and testing sets. A threshold of the LogR probability was chosen using the maximum Youden’s J statistic to classify “heal” and “not heal” pixels. The general models were generated using data from all four animals and applied to every pixel within the full-FOV CDD map derived from the MLR model. The results from feature-reduced models were again compared with those generated using all 40 features.

The LogR model used only histology-derived CDD as the predictor and healing outcome as the response. The FS methods described above therefore did not modify the LogR model itself. However, the CDD maps produced by the FS-MLR models differ from the baseline, leading to variations in the LogR output, as shown in Sec. 4.3.

## 4 Results

### 4.1 Feature-Selected Multiple Linear Regression Achieved Baseline-Comparable Performance with 13 LASSO Features and an Eight-Feature MFAE Subset

The three FS methods produced unique rank-ordered lists of features, as detailed in Tables 2–4 in Appendix B. Using OLS with the 13 most important LASSO features resulted in the minimum mean RMSE of 98  μm across all four folds of LOSO CV [Fig. 4(a)], achieving the best performance among all tested models. In addition, SOLS and mRMR achieved local minimum mean RMSE values by employing 12 and 26 features, respectively. The reduced number of features highlights the redundancy in the predictor variable space. The mean adjusted $R^{2}$ across all LOSO CV folds for the three FS methods at their respective optimal performance points is comparable to the value of 0.71 for the all-feature model [Fig. 4(b)].

**Fig. 4:**
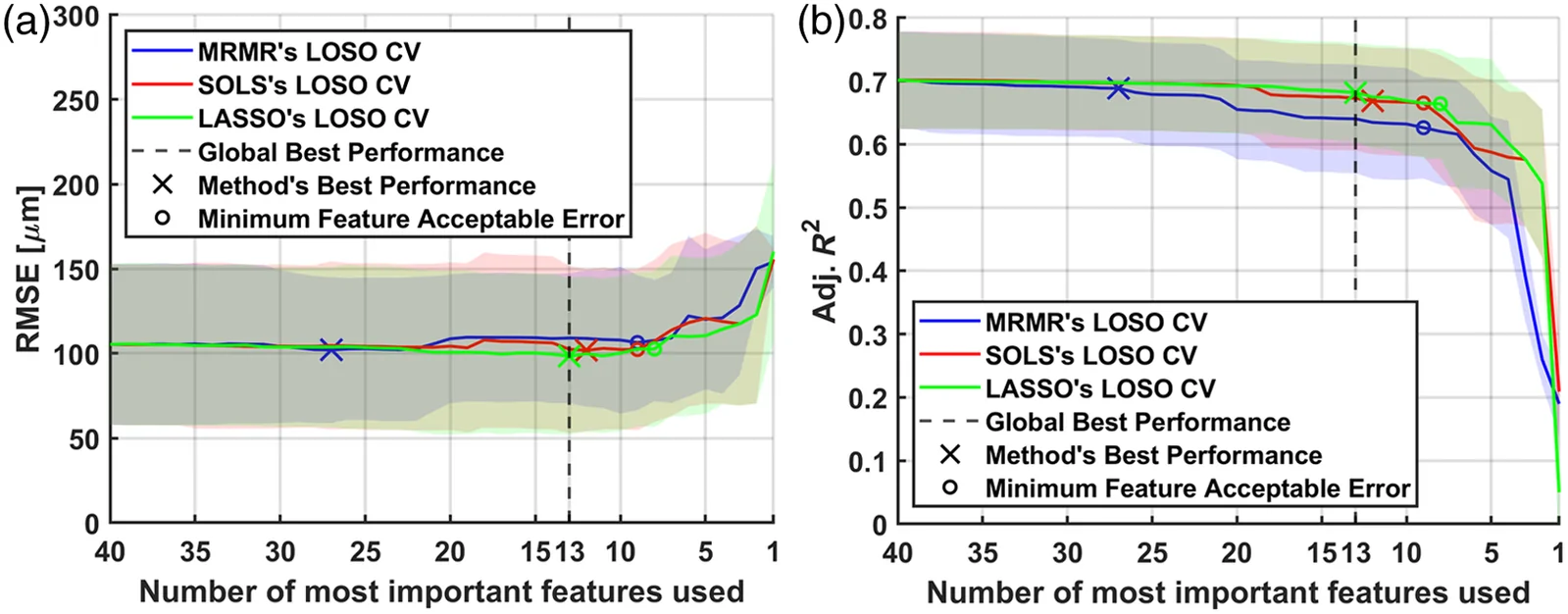
LOSO CV performance of OLS across the three FS methods with an increasing number of important features used. (a) The mean root mean squared error (RMSE) indicates that LASSO with 13 features achieves the best performance, whereas eight features represent the minimum features yielding acceptable performance (MFAE). (b) The adjusted $R^{2}$ of both the best-performing and MFAE models closely approximates the baseline model with 40 features. The solid lines represent the mean across all LOSO folds, whereas the shaded regions indicate the standard deviation.

We further identified the minimum feature set with acceptable error (MFAE) using a 5-micron tolerance (∼5% of the best-performance RMSE). The MFAE point for LASSO consists of eight features, whereas both SOLS and mRMR consist of nine features [Fig. 4(a)]. The mean RMSE ranged from ∼100 to ∼150  μm for all three FS methods [Fig. 4(a)]. Section 5 discusses the source of this error range, a potential approach to provide better performance, and the model’s practicality in burn wound assessment.

We applied the general model to individual pixels to generate full-FOV maps. Using the outputs of the 40-feature model as the baseline, we calculated the differences for each feature-reduced model. Specifically, we defined |ΔCDD| as the absolute pixel-wise difference between the model of interest and the baseline 40-feature model. Visually, we observed that both the best-performance 13-feature and the MFAE eight-feature models yielded |ΔCDD|<100  μm [Figs. 5(a) and 5(b)]. Quantitatively, both the best-performance and the MFAE models yielded a mean difference of <20  μm from the baseline 40-feature model [Fig. 5(c)].

**Fig. 5:**
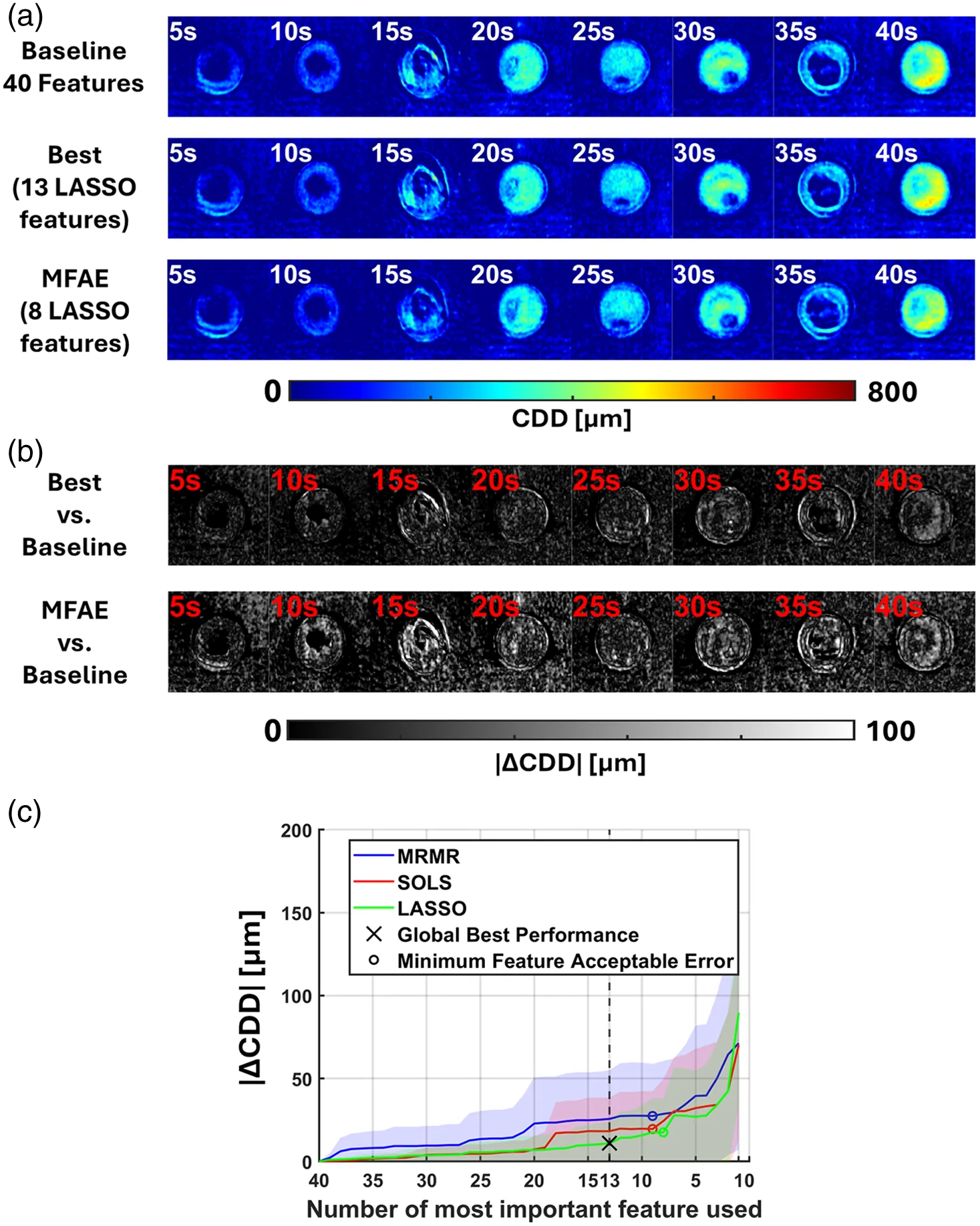
Application of the general MLR models to generate full-FOV collagen denaturation depth (CDD) maps and their comparison to the baseline 40-feature model. (a) CDD maps of the best-performance, the minimum features with acceptable error (MFAE), and the baseline 40-feature models show minimal differences. (b) Both best-performance and MFAE models showed a difference (|ΔCDD|) of less than 100  μm for most pixels within the FOV when compared with the baseline 40-feature model. (c) Both the best-performance 13-feature and MFAE eight-feature models yielded a mean CDD difference (|ΔCDD|) of <20  μm from the baseline 40-feature model. The solid line represents the mean value of all pixels, whereas the shaded region indicates the standard deviation.

### 4.2 Feature-Reduced Logistic Regression Models Yielded Similar Predictive Capabilities as That of the Baseline 40-Feature Model

As the LogR model was generated using only day-1 CDD as input and day-28 healing outcome as output, the model’s performance based on data chosen within the ROI remains unaffected. Recall that the LogR model achieved a mean ROC AUC of 0.81 for training data and 0.88 for testing data across all LOSO CV folds.^25^ Furthermore, the general model achieved an AUC of 0.80 with a maximum Youden’s J statistic of 0.55, which was selected as the threshold for classifying healing outcomes (“heal” versus “not heal”). This statistic corresponds to a healing probability threshold of 0.31 and ∼181  μm in CDD.^25^

In the current study, we evaluated differences in performance among the FS methods by applying the general LogR model to individual pixels to generate full-FOV maps of day-28 healing-outcome probability and classification. Compared with the baseline 40-feature model, both MFAE and best-performance models yielded minimal differences in probability and classification maps [Figs. 6(a) and 6(b)].

**Fig. 6:**
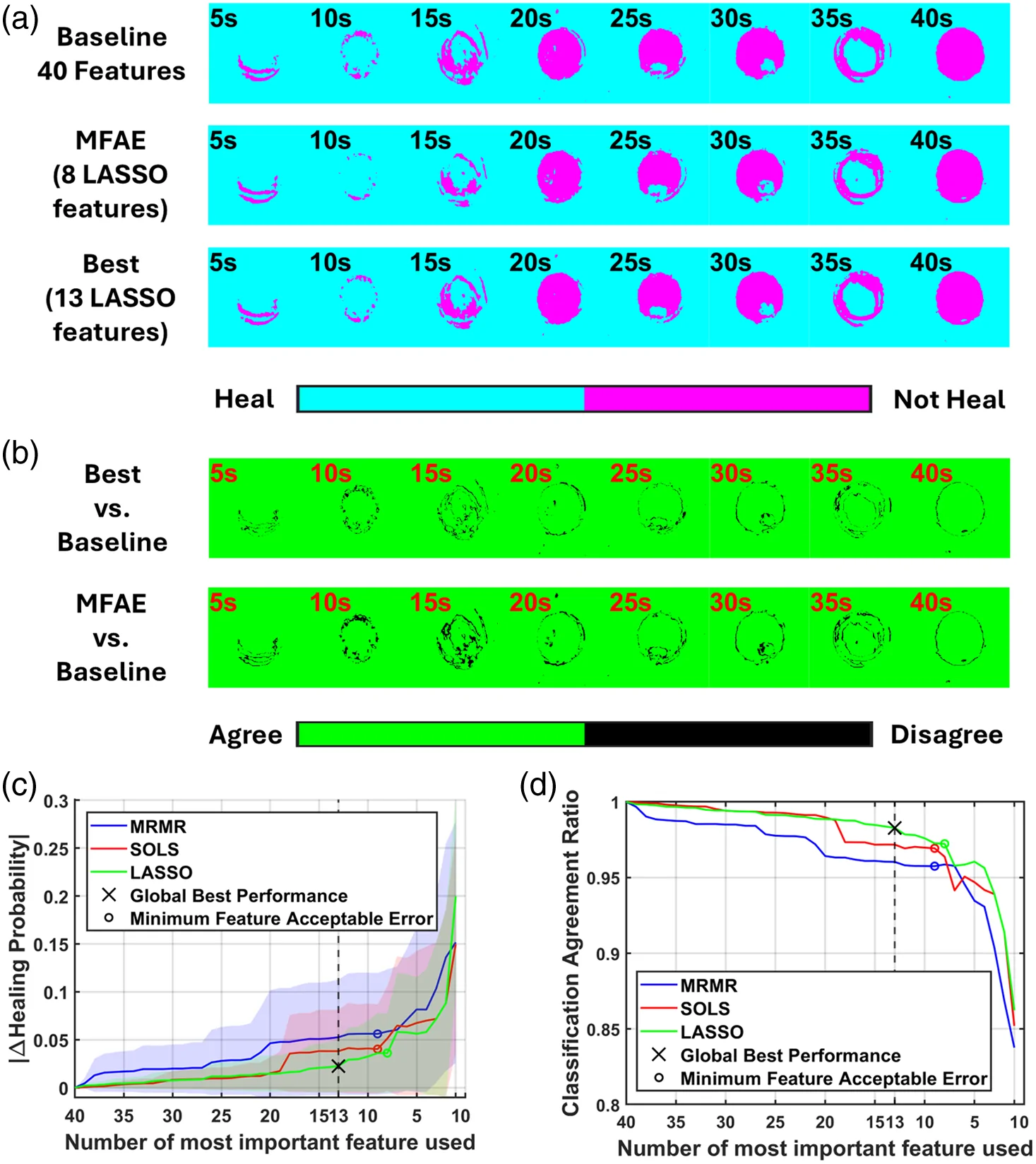
Application of the general LogR model to generate full-FOV day-28 healing outcome classification maps and their comparison to the baseline 40-feature model. (a) MFAE and best-performance maps of day-28 healing outcome showed minimal differences from those of baseline 40-feature model. (b) When compared with the baseline 40-feature model, the best-performance and MFAE models showed great agreement in classification. (c) and (d) For LogR, both the best-performance and MFAE models yielded a mean healing probability difference of <0.05 and a classification agreement ratio of >0.95 when compared with the baseline 40-feature model. For panel (c), the solid line represents the mean value of all pixels, whereas the shaded region indicates the standard deviation.

For quantitative analysis, we defined |ΔHealing Probability| as the absolute difference between the FS model of interest and the baseline 40-feature model. Similarly, we calculated a classification agreement ratio between each FS models and the baseline 40-feature model. Both best and MFAE models showed a mean |ΔHealing Probability| value of 0.05 [Fig. 6(c)]. The classification agreement ratio remained above 0.95 for both models compared with the baseline outputs [Figs. 6(d)].

### 4.3 Ranked Lists of Features by Importance Quotients for Each Feature Selection Method

Each FS method yielded a ranked list of SFDI features by their respective IQ. An IQ of 1 refers to the most important feature, and a fractional IQ indicates a ratio of feature importance to that of the most important feature. The 10 most important features of each method are listed in Table 1. Heatmaps and full lists of each feature and their respective IQs can be found in Figs. 8–10 in Appendix B.

**Table 1:** List of the 10 most important SFDI features identified by LASSO, mRMR, and SOLS in a descending order (i.e., 1 being the most important) and their respective wavelength and spatial frequency.

Importance rank (descending from most importance)	LASSO		mRMR		SOLS	
	Wavelength (nm)	Spatial frequency (${\mathrm{mm}}^{-1}$)	Wavelength (nm)	Spatial frequency (${\mathrm{mm}}^{-1}$)	Wavelength (nm)	Spatial frequency (${\mathrm{mm}}^{-1}$)
1	526	0	851	0	621	0
2	621	0	591	0	526	0
3	526	0.15	526	0	526	0.15
4	471	0.2	659	0	591	0
5	691	0.1	691	0.2	591	0.05
6	471	0	621	0	471	0.1
7	591	0	691	0	621	0.15
8	851	0.05	659	0.2	471	0.2
9	691	0.05	591	0.1	591	0.2
10	691	0	731	0.1	691	0.1

## 5 Discussion

### 5.1 Summary of Methods and Notable Findings

In this study, we refined a previously developed two-step regression framework that used day-1 SFDI $R_{d}$ maps to predict same-day CDD and, ultimately, day-28 healing outcomes. We incorporated three feature selection (FS) techniques—SOLS, LASSO, and mRMR—to identify key SFDI features for assessing CDD, reducing the model from the original 40 features while maintaining performance.

Our results showed that a model incorporating the 13 most important LASSO features achieved the lowest mean RMSE for predicted CDD across all LOSO CV folds, indicating the best MLR performance. In addition, the MFAE model using eight LASSO features closely approximated the performance of the 13-feature model, with a mean RMSE difference of ∼5  μm (or 5%). Both the MFAE and best-performance models demonstrated results comparable to the 40-feature baseline, less than 20  μm, underscoring the redundancy among features. It should be noted that all three feature-selection methods achieved similar performance when using their respective optimal sets of SFDI features. Numerically, LASSO with 13 features produced the best results, exhibiting the lowest RMSE [Fig. 4(a)], highest $R^{2}$ [Fig. 4(b)], and lowest |ΔCDD| [Fig. 5(c)]. However, this performance advantage is marginal, and any of the three methods can be used for feature reduction. For the LogR step, both the best-performing and MFAE models closely matched the baseline 40-feature model, yielding a mean healing probability difference of less than 0.05 and a classification agreement ratio exceeding 0.95 across all pixels within the full FOV.

Regarding the impact of reducing the dataset, the underlying model is a straightforward multiple linear regression (MLR). Consequently, decreasing the feature count from 40 to 8 did not meaningfully affect model training or utilization due to limited sample size. All wavelengths and spatial frequencies were acquired using the three-phase demodulation method; thus, reducing the number of features would translate to a linear reduction in acquisition time and processing load. Practical hardware considerations, such as exposure time, also play a role. CMOS cameras typically exhibit higher sensitivity in the visible range, with peak sensitivity in the green region. Therefore, having most of the important features located in the green wavelengths would further reduce acquisition time.

### 5.2 Interpreting Feature Importance in Predicting CDD

This section examines the ranking of key features identified by the three FS methods as listed in Table 1. Three features are consistently ranked among the top 10 most important across all three FS methods: $0\;{\mathrm{mm}}^{-1}$ at 526 nm, $0\;{\mathrm{mm}}^{-1}$ at 591 nm, and $0\;{\mathrm{mm}}^{-1}$ at 621 nm. LASSO and SOLS share three additional features: $0.2\;{\mathrm{mm}}^{-1}$ at 471 nm, $0.15\;{\mathrm{mm}}^{-1}$ at 526 nm, and $0.1\;{\mathrm{mm}}^{-1}$ at 691 nm. Meanwhile, LASSO and mRMR share one feature: $0\;{\mathrm{mm}}^{-1}$ at 691 nm. Notably, higher spatial-frequency features at 471, 526, and 691 nm appear in two of the FS methods.

In SFDI, lower spatial frequencies are more sensitive to absorption changes, whereas higher spatial frequencies are more sensitive to scattering changes.^16^^,^^17^^,^^41^ The existence of higher spatial frequencies within the 10 most important features suggests that scattering changes play a key role in determining CDD. The presence of both low and high spatial frequencies at specific wavelengths, such as 526 nm, indicates that the distinction between superficial and deep tissue, such as denatured versus healthy collagen matrix, may contribute to tracking CDD.^26^^,^^28^^,^^42^ Interestingly, Ritz et al. reported that coagulated blood in a porcine liver showed a difference in absorption coefficients and an increase in reduced scattering coefficient.^43^ However, this scattering increase from coagulated blood is mostly negligible compared with the reduction caused by denatured collagen due to the small blood volume fraction in the skin. Furthermore, the recurring prominence of planar reflectance at 526, 591, and 621 nm suggests that blood coagulation or breakdown byproducts, such as methemoglobin, and its associated absorption changes, may be a contributing factor. This may be due to CDD’s strong correlation with actual burn depth, which is partially assessed by examining coagulated blood vessels.^44^^,^^45^

### 5.3 Study Limitations and Future Directions

Moving forward, we must validate these optimized feature sets across larger and more diverse subject cohorts to ensure that the identified visible-light biomarkers remain robust against the confounding effects of melanin. It is worth noting that although visible wavelengths are highly predictive in the current porcine model, which has minimal epidermal melanin, future studies in subjects with darker skin tones may reveal that near-infrared wavelengths (e.g., 851 nm) provide greater generalizability for burn-wound assessment.^18^ From our experience, the epidermis is often damaged and absent in thermal burns; consequently, the melanin layer may be unavailable for imaging. Nevertheless, to achieve true real-time clinical utility, these reduced feature sets could be integrated with specialized hardware, such as multispectral CMOS image sensors,^46^ which allows for simultaneous multiwavelength acquisition and improved suppression of ambient light bias, both of which are important factors when imaging in a surgical theater or on the field.

Second, this study employed SOLS, LASSO, and mRMR for feature selection, but these methods are not comprehensive. Alternative approaches could be explored, such as ridge regression, elastic-net regression, neighborhood component analysis, and F-tests for regression. For classification, logistic regression was the only method tested; however, other methods, including support vector machine, nearest neighbors, and naïve Bayes, offer potential for further investigation. In addition, future studies focusing on classifying burns into clinical burn depth categories can benefit from using feature selection methods tailored to classification models, such as neighborhood component analysis, mRMR, and Chi-square tests.

Last, although LASSO identified a reduced set of eight viable SFDI features, the resulting combination of wavelengths and spatial frequencies proves challenging to implement in a compact imaging system (i.e., different spatial frequencies at various wavelengths). Because LASSO tends to remove collinear features, a dedicated collinearity analysis could reveal alternative SFDI features with shared wavelengths that would simplify system design. Furthermore, analyzing interaction terms (e.g., products or ratios of features) would help identify and solidify the minimum set of SFDI features required for reliable burn-wound assessment. This represents an important direction for future work, enabling further system optimization and development of a compact system. Such a compact and mobile system would enable direct clinical translation, especially in far-forward scenarios such as deployment in austere environments and mass-casualty settings.

## 6 Conclusion

This investigation establishes that the data complexity inherent to high-fidelity spatial frequency domain imaging (SFDI) can be significantly streamlined without sacrificing the predictive accuracy required for burn triage. By applying feature selection methods (LASSO, MRMR, and SOLS) to our established two-step regression framework, we identified a high-performance subset of 13 features that achieved a mean RMSE of 98  μm for predicting collagen denaturation depth (CDD). This approximately matches the 105  μm RMSE reported in our preceding work, which utilized the full 40-feature baseline.^25^ Furthermore, a minimalist set of only eight LASSO features proved sufficient to maintain a ≥0.95 classification agreement ratio for predicting day-28 healing outcomes compared with the 40-feature baseline model. The consistent ranking of planar reflectance at 526, 591, and 621 nm among the top diagnostic features underscores the critical importance of capturing hemoglobin-related absorption changes alongside structural scattering shifts when assessing the depth of thermal injury. Ultimately, this 80% reduction in feature count provides a viable path toward a compact SFDI system. As discussed in Sec. 5.3, the present LASSO subset combines several spatial frequencies across different wavelengths and is not yet directly translatable to a maximally simplified acquisition; a future collinearity analysis to identify wavelength-aligned feature sets will be required before claims of reduced acquisition time and improved motion-artifact tolerance can be substantiated.

## 7 Appendix A: Mathematical Terminology, Notations, and Definitions

### 7.1 Standardization

In statistics, standardization refers to the process of normalizing data using an assumption of the data distribution. In this study, we standardized the input data using z-score as defined below:

$$z=\frac{x-\overset{¯}{x}}{\mathrm{std}(x)}.$$

z is the z-score or standardized data, x is the input data, $\overset{¯}{x}$ is the mean of the input data, and std(x) is the standard deviation of the input data. Here, we assume that the data follow a normal distribution. Thus, standardization recenters and scales the distribution to a mean of zero and a standard deviation of 1. In the context of linear regression as discussed below, this allows for a direct comparison of the contribution of each predictor variable based on the accompanying regression coefficient β. In this study, we implemented standardization with MATLAB’s “normalize” function.

### 7.2 Multiple Linear Regression

MLR is a method for deriving a linear equation that describes the relationship between multiple predictor variables and a single response variable.

### 7.3 Ordinary Least Squares and Standardized Ordinary Least Squares

Ordinary least squares (OLS) is a method of finding regression coefficients that achieve the best-fit MLR line by minimizing the square error of the predicted response variables. The mathematical definition of the optimization problem for N observations with P features is as follows:

$${\arg\;\min}_{\beta }(\overset{N}{\underset{j=1}{\sum }}{{\left(y-\overset{P}{\underset{i=1}{\sum }}\beta_{i}x_{i}-\beta_{0}\right)}_{j}}^{2}\;).$$

β is the regression coefficient, x is the predictor variable, y is the response variable, $\beta_{0}$ is the y-intercept,  i is the feature index, and j is the observation index. In this study, “feature” refers to the SFDI-calibrated reflectance at a known spatial frequency and wavelength. We implemented OLS for MLR with MATLAB’s “regress” function.

In OLS, regression coefficients β can indicate how strongly each predictor contributes to the response. However, when predictors differ in scale or distribution, coefficients can be misleadingly inflated simply due to differences in magnitude. To place all predictors on a comparable scale, standardization is typically applied, yielding standardized ordinary least squares (SOLS).

### 7.4 Least Absolute Shrinkage and Selection Operator

Least absolute shrinkage and selection operator (LASSO) regularization is another method of achieving the best-fit MLR line while attempting to minimize the contribution of redundant predictor variables. LASSO achieves this by adding a penalty term to the minimization argument that accounts for the size of the regression coefficients. The mathematical definition of the optimization problem for N observations with P features is as follows:

$${\arg\;\min}_{\beta }(\overset{N}{\underset{j=1}{\sum }}{{\left(y-\overset{P}{\underset{i=1}{\sum }}\beta_{i}x_{i}-\beta_{0}\right)}_{j}}^{2}+\lambda \overset{P}{\underset{i=1}{\sum }}|\beta_{i}|).$$

β is the regression coefficient, x is the predictor variable, y is the response variable, $\beta_{0}$ is the y-intercept, i is the feature index, and j is the observation index. Here, the equation introduces the bias term of a sum of all β values with a regularization factor λ. The optimal regularization factor λ is determined through k-fold cross-validation (CV). In this study, λ was determined as one standard error away from the point of minimum mean squared error (minMSE) across all CV folds. We implemented LASSO with MATLAB’s “lasso” function with an “Alpha” value of 1. Unlike OLS, LASSO will reduce the regression coefficients to zero for irrelevant predictor variables. In addition, LASSO is more resilient than OLS in the presence of extreme outliers.

### 7.5 Minimum Redundancy Maximum Relevance

Minimum redundancy maximum relevance (mRMR) is a modern feature selection method that relies on mutual information among the existing variables.^39^ Here, mutual information is defined as

$$I(A,B)=\underset{i,j}{\sum }P(A=a_{i},B=b_{i})\log(\frac{P(A=a_{i},B=b_{i})}{P\left(A=a_{i}\right)P(B=b_{i})}).$$

$P(A=a_{i},B=b_{i})$ is the joint probability mass function of variables A and B, $P(A=a_{i})$ and $P(B=b_{i})$ are the marginal probability mass functions of variables A and B, respectively, and i and j are observation index of A and B. The implementation of such equation on continuous variables can be achieved by discretizing such variables into bins. MATLAB’s implementation (“fsrmrmr”) uses 256 bins when the number of unique values exceeds this threshold, which applies in this work due to the pixel-level sampling of reflectance.

In mRMR, data relevance $V_{x}$ is defined as the mutual information between each predictor and response variable. Meanwhile, redundancy $W_{x}$ is defined as the mutual information between two predictor variables. Then, mRMR ranks the features (i.e., predictor variables) by defining the mutual information quotient MIQ as:

$$\mathrm{MIQ}=\frac{V_{x}}{W_{x}}.$$

For interpretation, predictor variables with higher MIQ are more uniquely relevant in predicting the response variable and thus is more important. We implement mRMR for regression with MATLAB’s “fsrmrmr” function.

### 7.6 Bootstrapping

In statistics, “bootstrapping” refers to the practice of subsampling the available samples to provide a more robust test statistic or a more stable model. In bootstrapping, subsampling occurs with replacement, where each datapoint can be sampled more than once at random with equal chance. It is often assumed that the sample distribution follows the population distribution, and thus, subsampling at random with replacement would draw samples representing such a distribution.

### 7.7 Root Mean Square Error

We use the root mean square error (RMSE) of the predicted response variable to evaluate the performance of the linear models. RMSE is calculated for N observation as follows:

$$\mathrm{RMSE}=\sqrt{\frac{1}{N}\sum (y_{p}-y_{t}{)}^{2}}.$$

$y_{p}$ is the predicted response variable, and $y_{t}$ is the observed true response variable. In this study, RMSE is calculated by assessing errors of the complete model and not just the fitted line. This means that RMSE calculation will account for the nonnegative boundary condition as discussed below. It should be noted that RMSE has the same unit as the response variable.

### 7.8 Adjusted Coefficient of Determination (Adjusted *R*^2^)

We assess the linear fit using the adjusted coefficient of determination (Adj. $R^{2}$). The Adj. $R^{2}$ is calculated for N observations and N features as follows:

$$\mathrm{Adj}.\;R^{2}=1-(\frac{N-1}{N-P})\frac{\sum {\left(y_{p}-y_{t}\right)}^{2}}{\sum {\left(y_{t}-\overset{¯}{y_{t}}\right)}^{2}}.$$

$y_{p}$ is the predicted response variable, and $y_{t}$ is the observed true response variable. Unlike the unadjusted $R^{2}$, which typically increases with increasing number of predictor variables, the Adj. $R^{2}$ assesses the model’s fitting performance regardless of the number of features used. This allows us to directly compare models following the feature selection step.

### 7.9 Nonnegative Boundary Condition

In this study, we chose collagen denaturation depth (CDD) as the response variable of our MLR models. Thus, the values of CDD should remain nonnegative. Nonnegative boundary conditions refer to the practice of limiting the lower bound of predicted CDD to zero. We employed this condition after achieving a fitted MLR model. Subsequently, RMSE is calculated after the application of the nonnegative boundary condition, whereas the Adj. $R^{2}$ is assessed before such application of the condition.

### 7.10 Logistic Regression

Logistic regression (LogR) is a simple classification algorithm that utilizes fitting a log-based link function to a binary class A to generate a probability curve. In this study, we used the logit link function (i.e., $\log(\frac{\mu }{1-\mu })$ where μ is the probability of being classified as A). From the generated probability curve, a threshold can be chosen to classify input data points as A or not A. We implemented LogR with MATLAB’s “fitglm” function with the logit link function for a binomial distribution.

### 7.11 Receiver Operating Characteristic Curve and Youden’s J Statistic

Receiver operating characteristic (ROC) curves are used to assess the accuracy of classification algorithms. In the ROC curve, the true positive rate (i.e., sensitivity) is plotted against the false positive rate (i.e., 1—specificity). A perfect binomial distribution or probabilistic model yields an area under the curve (AUC) of 1. Furthermore, to determine an optimal threshold for the binary classification, one can determine a threshold that maximizes the Youden’s J Statistic defined as follows:

J=True Positive Rate−False Positive Rate=Sensitivity+Specificity−1.

In essence, maximizing Youden’s J Statistic finds the threshold that allows for the best balance of sensitivity and specificity. A Youden’s J value of 1 indicates a perfect model for both sensitivity and specificity, whereas a value of 0 indicates random chance. We implemented the ROC curve analysis with MATLAB’s “perfcurve” function.

### 7.12 Leave-One-Subject-Out Cross-Validation

In statistics and machine learning model development, cross-validation (CV) refers to the process of assessing the model’s performance by dividing the available data into training and testing sets. Leave-one-subject-out (LOSO) is a rigorous cross-validation method that treats the data from one subject as the testing set, whereas the rest of the data are used for training. For each fold of the process, LOSO CV uses independent data (i.e., different subjects) for testing and thus fully captures the model’s performance behavior. In this study, “subject” refers to one of the four animals. Following LOSO CV, we used the data from all subjects to generate the “general” model.

## 8 Appendix B: Supplementary Information

### 8.1 Feature Selection Pipelines

SOLS: In standard linear regression, assuming no extreme outliers and standardized data, the regression coefficients directly reflect each predictor variable’s contribution to the model’s response variable. We applied SOLS with the standardization of both predictor and response variables. Importance quotient (IQ) was defined as the ratio of each feature’s absolute mean coefficient to the largest absolute mean coefficient (Fig. 7—left). A ranked list of features in descending IQ order was then generated.

**Fig. 7:**
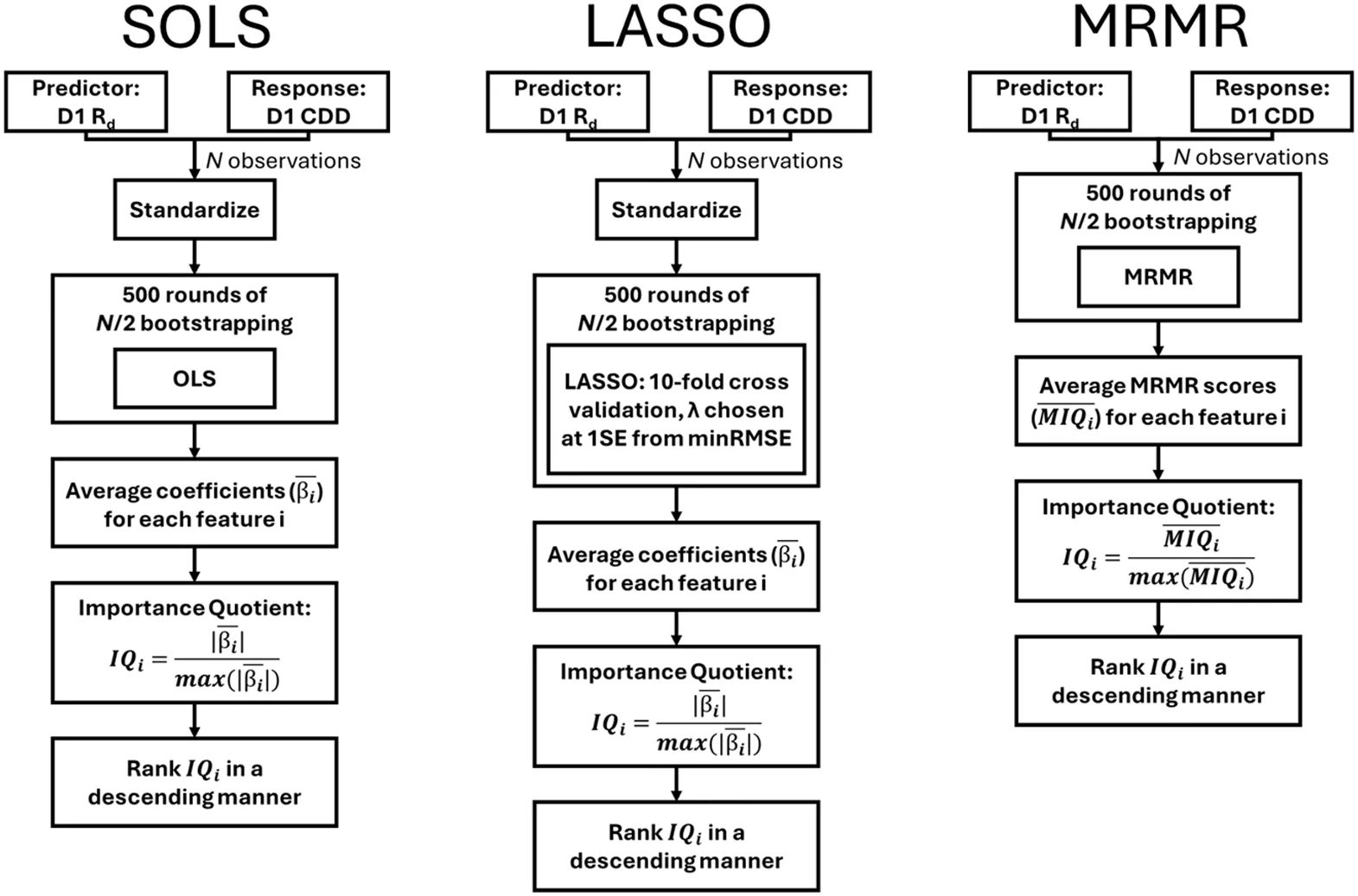
Overview of the three feature selection (FS) methods used in this study, along with their corresponding definitions of importance quotients (IQ).

LASSO: MLR with LASSO regularization was performed with standardized data to define IQ similar to that of SOLS (Fig. 7—center). The regularization factor was selected using 10-fold cross-validation. Finally, features were ranked in descending IQ order.

mRMR: Unlike SOLS and LASSO, mRMR evaluates feature importance using mutual information across all variables. We applied mRMR without standardization, calculating average scores for each feature. IQ was defined as the ratio of each feature’s absolute mean score to the highest absolute mean score (Fig. 7). Features were then ranked in descending IQ order. The resulting list of features in descending order of importance for each method can be found in Tables 2–4. Heatmaps of each table are also included in Figs. 8–10.

**Fig. 8:**
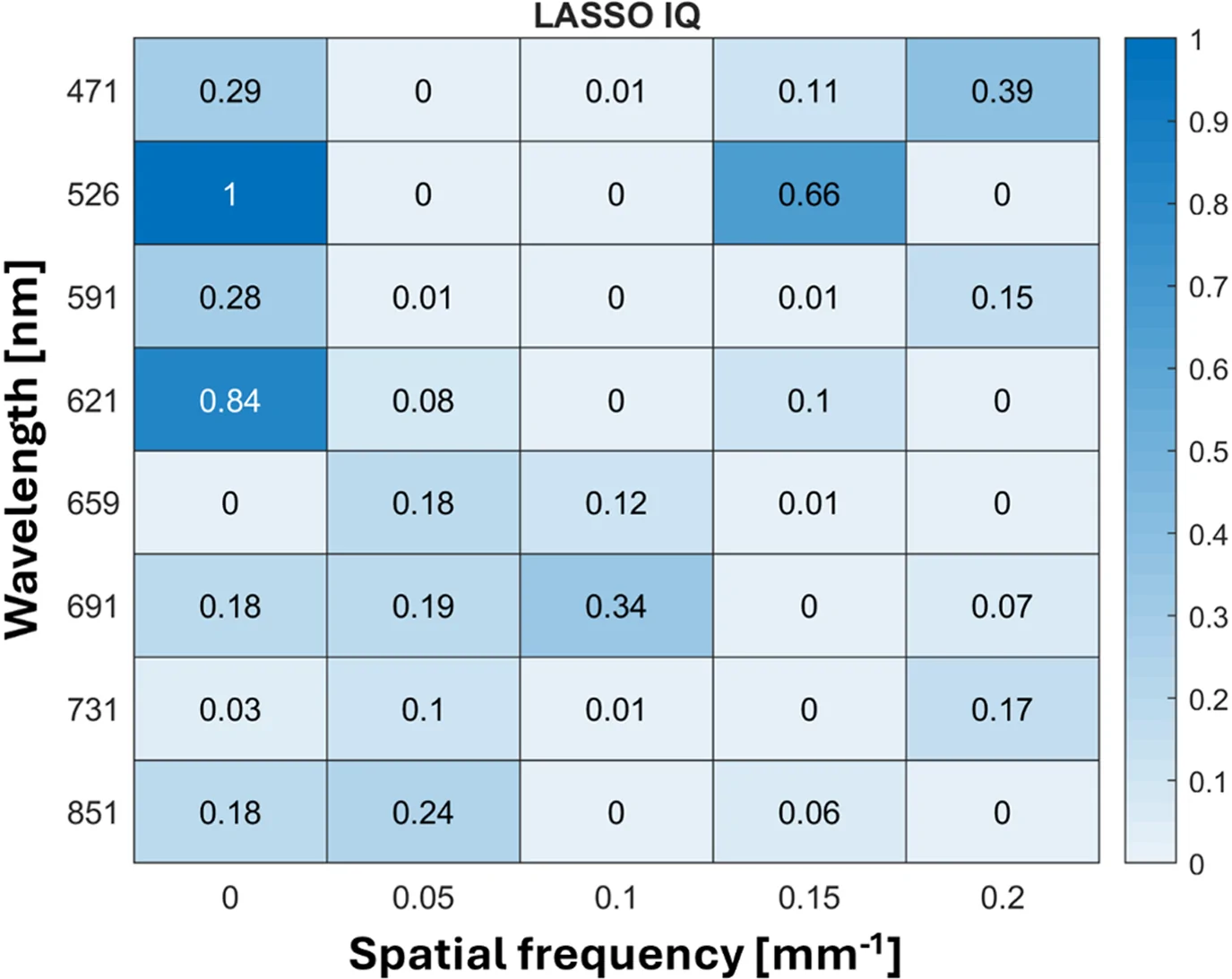
Heatmap of importance quotients (IQ) for all SFDI features, calculated using LASSO.

**Fig. 9:**
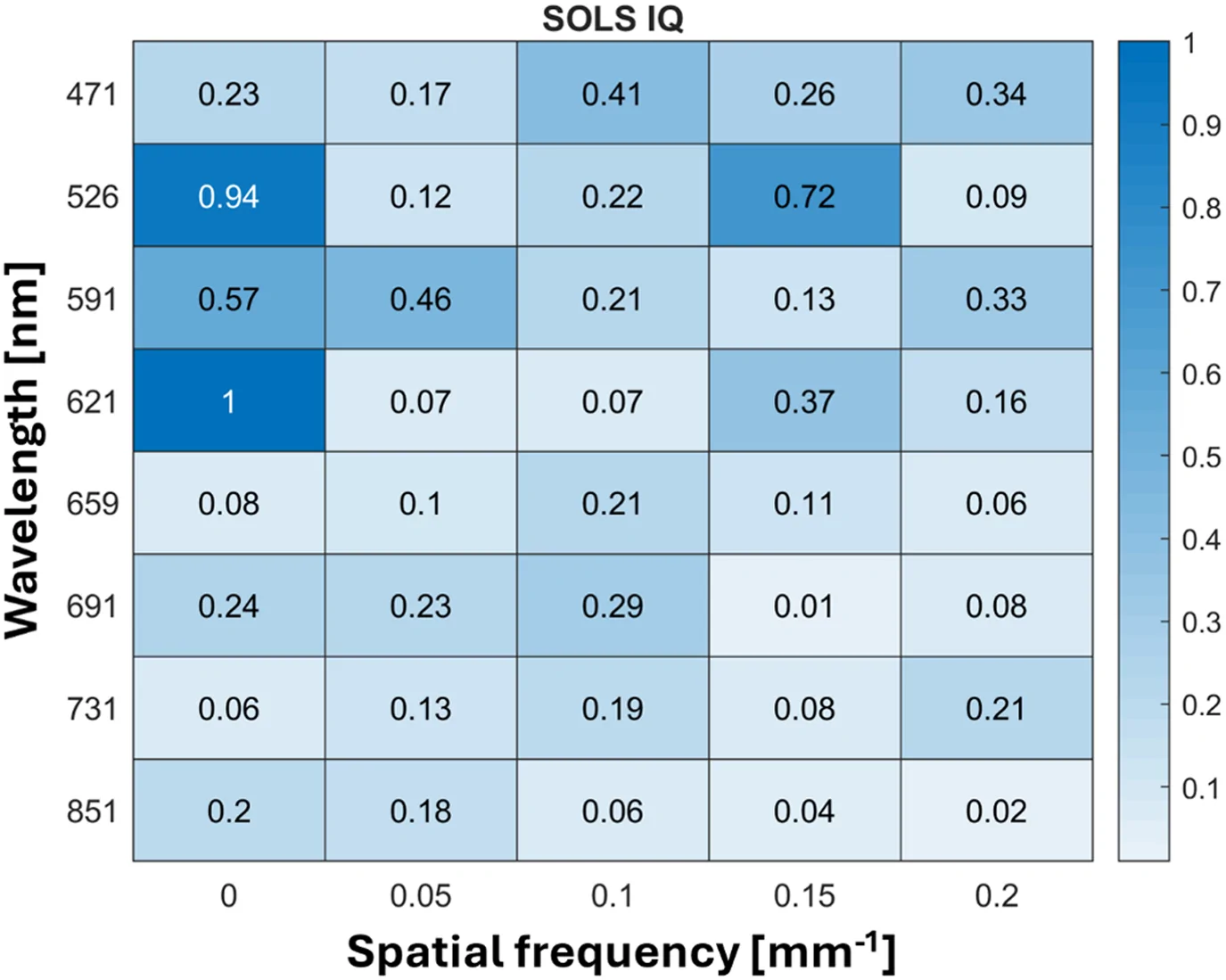
Heatmap of all SFDI features of importance quotients (IQ) calculated using SOLS.

**Fig. 10:**
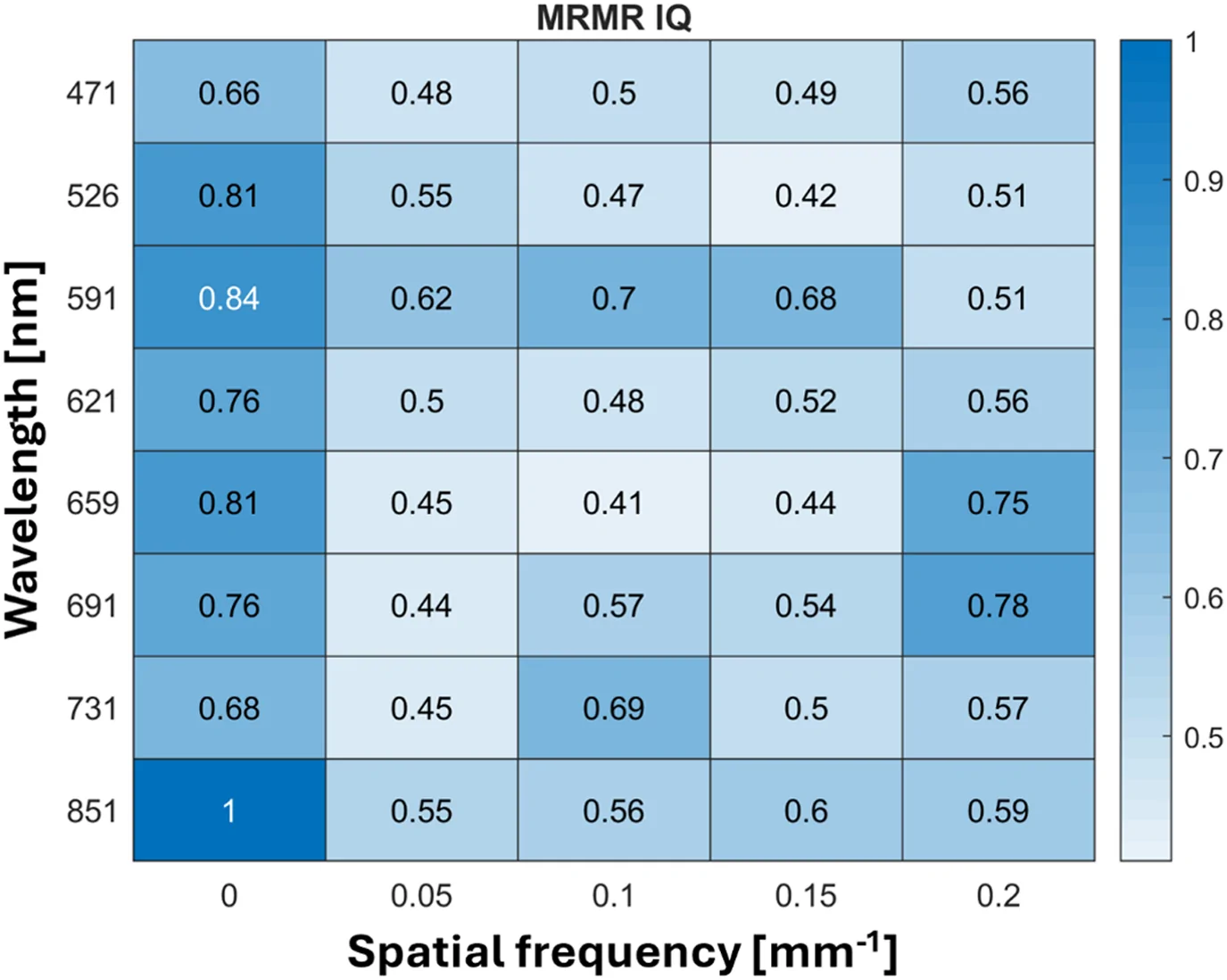
Heatmap of all SFDI features of importance quotients (IQ) calculated using mRMR.

**Table 2:** Feature list of LASSO feature selection and the accompanying IQ.

IQ	Wavelength (nm)	Spatial frequency (${\mathrm{mm}}^{-1}$)
1	526	0
0.83731	621	0
0.664409	526	0.15
0.392041	471	0.2
0.336231	691	0.1
0.291724	471	0
0.277502	591	0
0.24444	851	0.05
0.187673	691	0.05
0.182582	691	0
0.181806	659	0.05
0.176897	851	0
0.17159	731	0.2
0.14561	591	0.2
0.117918	659	0.1
0.105735	471	0.15
0.103343	621	0.15
0.099041	731	0.05
0.084783	621	0.05
0.067253	691	0.2
0.056011	851	0.15
0.034699	731	0
0.011093	471	0.1
0.008903	591	0.15
0.008472	731	0.1
0.008188	659	0.15
0.00514	591	0.05
0.004417	851	0.1
0.003348	851	0.2
0.003062	471	0.05
0.002922	691	0.15
0.002455	591	0.1
0.00143	621	0.2
0.000739	659	0.2
0.00055	526	0.2
0.000317	526	0.05
0.000216	526	0.1
0.000214	731	0.15
0	621	0.1
0	659	0

**Table 3:** Feature list of SOLS feature selection and the accompanying IQ.

IQ	Wavelength (nm)	Spatial frequency (${\mathrm{mm}}^{-1}$)
1	621	0
0.944155	526	0
0.71821	526	0.15
0.566781	591	0
0.455817	591	0.05
0.409284	471	0.1
0.371569	621	0.15
0.344349	471	0.2
0.332235	591	0.2
0.287142	691	0.1
0.257372	471	0.15
0.23564	691	0
0.234264	691	0.05
0.231517	471	0
0.221909	526	0.1
0.210442	659	0.1
0.206614	731	0.2
0.205327	591	0.1
0.200003	851	0
0.194246	731	0.1
0.182921	851	0.05
0.167029	471	0.05
0.162953	621	0.2
0.130213	731	0.05
0.125412	591	0.15
0.119067	526	0.05
0.105904	659	0.15
0.101263	659	0.05
0.091136	526	0.2
0.079664	731	0.15
0.07948	691	0.2
0.076628	659	0
0.069901	621	0.05
0.066731	621	0.1
0.058597	659	0.2
0.05578	731	0
0.055197	851	0.1
0.039808	851	0.15
0.021152	851	0.2
0.011566	691	0.15

**Table 4:** Feature list of mRMR feature selection and the accompanying IQ.

IQ	Wavelength (nm)	Spatial frequency (${\mathrm{mm}}^{-1}$)
1	851	0
0.843038	591	0
0.8071	526	0
0.806411	659	0
0.778478	691	0.2
0.758666	621	0
0.757506	691	0
0.753986	659	0.2
0.696799	591	0.1
0.689828	731	0.1
0.683524	731	0
0.682872	591	0.15
0.658041	471	0
0.619676	591	0.05
0.604887	851	0.15
0.592261	851	0.2
0.567701	731	0.2
0.566824	691	0.1
0.563781	851	0.1
0.560736	621	0.2
0.560255	471	0.2
0.552918	526	0.05
0.546686	851	0.05
0.535085	691	0.15
0.517558	621	0.15
0.506469	591	0.2
0.506455	526	0.2
0.504738	731	0.15
0.499534	471	0.1
0.499133	621	0.05
0.494389	471	0.15
0.477551	621	0.1
0.475053	471	0.05
0.470447	526	0.1
0.447906	731	0.05
0.446417	659	0.05
0.442634	659	0.15
0.44115	691	0.05
0.422735	526	0.15
0.40684	659	0.1

## Data Availability

The datasets generated and/or analyzed during the current study are available upon reasonable request. The project is currently hosted on Zenodo: https://doi.org/10.5281/zenodo.22858549.
